# Linking Epidemiology and Whole-Genome Sequencing to Investigate *Salmonella* Outbreak, Massachusetts, USA, 2018

**DOI:** 10.3201/eid2607.200048

**Published:** 2020-07

**Authors:** Eric L. Vaughn, Quynh T. Vo, Johanna Vostok, Tracy Stiles, Andrew Lang, Catherine M. Brown, R. Monina Klevens, Lawrence Madoff

**Affiliations:** Massachusetts Department of Public Health, Boston, Massachusetts, USA

**Keywords:** Salmonella, foodborne illness, whole-genome sequencing, bacteria, Massachusetts, United States, epidemiology, food safety

## Abstract

Cross-discipline collaboration among state and local health departments improved foodborne illness surveillance for a 2018 *Salmonella enterica* serovar Enteritidis outbreak in Massachusetts, USA. Prompt linking of epidemiologic and laboratory data and implementation of in-state whole-genome sequencing and analysis improved public health surveillance capacity for outbreak detection and control.

Nontyphoidal salmonellae are among the most ubiquitous pathogens associated with foodborne illness worldwide (*1*). Salmonellosis detection is of public health value because of the ease with which the pathogen is transmitted (*2*), increased pathogen antimicrobial drug resistance (*3*), and high morbidity rates (*4*). Detection of *Salmonella* in a Massachusetts resident is reportable to the Massachusetts Department of Public Health (MDPH), and submission of isolates to the MDPH State Public Health Laboratory (SPHL) is mandatory. From 2014 through 2018, an average of 1,200 confirmed cases of *Salmonella* infection in Massachusetts residents were reported each year. Since 1996, MDPH has used pulsed-field gel electrophoresis (PFGE) to identify local clusters and report isolate patterns to the Centers for Disease Control and Prevention (CDC) PulseNet network (https://www.cdc.gov/pulsenet/index.html). Increasingly, public health laboratories are using whole-genome sequencing (WGS) to identify unique strains and outbreaks. MDPH began using WGS in 2015 and fully transitioned to use of this technique in 2019.

After sequencing is complete, isolates are further analyzed by using a reference-free bioinformatics pipeline, a system first developed in Utah (*5*) and optimized by an MDPH bioinformatician for the Massachusetts SPHL. This pipeline enables creation of a phylogenetic tree, which shows relatedness between isolates. Concurrent with the analysis and development of the tree at the SPHL, the raw sequencing data (FastQ files) are uploaded to PulseNet for core genome multilocus sequence typing analysis to determine allele differences between the isolates. In 2018, a total of 583 of 803 *Salmonella* isolates underwent sequencing in Massachusetts and results were sent to CDC for confirmatory analysis.

Massachusetts uses an integrated web-based surveillance and case-management system for >90 reportable infectious diseases, including *Salmonella* (*6*). After a case is received through electronic laboratory reporting, local boards of health are notified about the need for case investigation. Clusters identified through laboratory or epidemiologic methods receive additional follow-up. The SPHL and Division of Epidemiology are part of the Bureau of Infectious Disease and Laboratory Sciences at MDPH, and these programs meet regularly to review clusters of infectious disease.

## The Study

In mid-October 2018, the SPHL notified epidemiologists about an increased number of isolates that were indistinguishable from *Salmonella enterica* serovar Enteritidis pattern JEGX01.0004, according to by PFGE testing over a 60-day period. The average baseline for this pattern is 12.5 isolates/year from August 15 through October 15; by October 15, 2018, a total of 34 isolates had been identified. Pattern JEGX01.0004 is the most common *Salmonella* Enteritidis pattern found in Massachusetts, making cluster detection difficult. All JEGX01.0004 isolates received priority status for WGS.

Before WGS data were available, epidemiologists identified 3 persons with laboratory-confirmed *Salmonella* infection who had been interviewed by the local board of health and reported having dined at the same restaurant on November 2, 2018. All 3 case-patients lived in adjacent towns in Middlesex County and had not dined together. Two had eaten chicken Caesar salads and 1 had eaten a Greek salad with chicken. All 3 were female and 50–70 years of age. Epidemiologists notified the SPHL of the epidemiologic link on November 15. On November 16, the laboratory confirmed that isolates from all 3 case-patients were indistinguishable from *Salmonella* Enteriditis pattern JEGX01.0004.

Subsequently, the MDPH Food Protection Program notified the local board of health for the implicated restaurant, which led to a same-day inspection of the establishment. The inspection noted several food safety issues, including lack of an employee illness plan and absence of a separate sink for handwashing. The restaurant closed for cleaning on November 17 and again on November 19 while fecal samples from the food handlers were screened for *Salmonella*. Of the 11 employees who submitted fecal samples to the SPHL for *Salmonella* testing, results were positive for 1 food handler on November 23. This person had been working at the restaurant on the weekend of November 2–4, the reported exposure dates for the first case-patients. During November 19–26, WGS added an additional 6 cases to the cluster.

While the initial investigation of the restaurant was under way, the laboratory used in-house sequencing analysis to generate a phylogenetic tree; CDC performed confirmatory analyses on the accompanying FastQ files. The information resulted in identification of 7 individual clusters within isolates with the PFGE pattern JEGX01.0004. Further epidemiologic investigations linked 1 cluster to the restaurant cluster; the other 6 clusters could not be linked to a common exposure (Figure).

**Figure:**
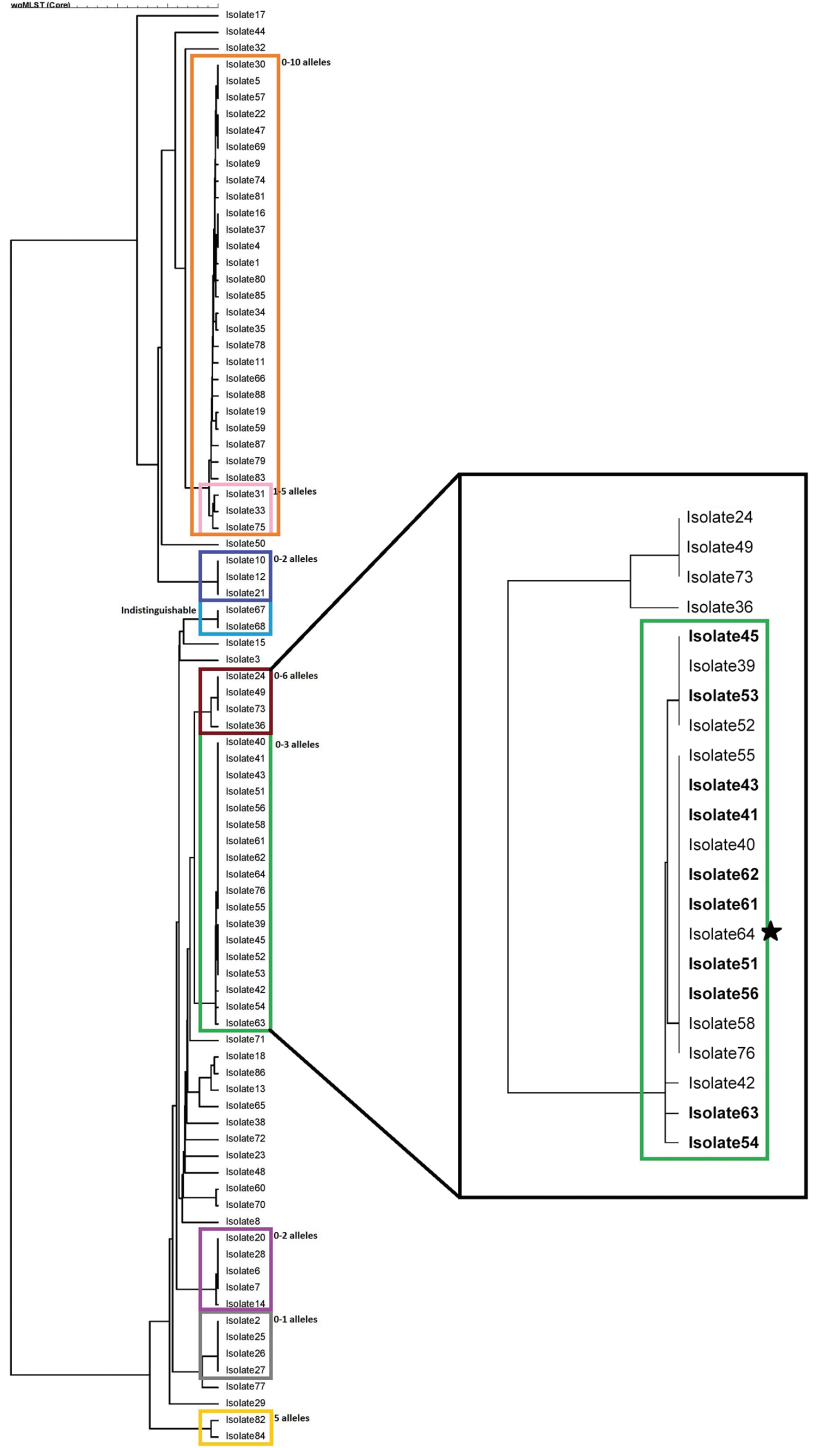
Phylogenetic tree for *Salmonella enterica* serotype Enteritidis isolates from outbreak in Massachusetts, USA, 2018. The colored boxes on the left indicate 9 separate subclusters for the entire 84-isolate cluster, with confirmation of allele differences coming from PulseNet (https://www.cdc.gov/pulsenet/index.html). Eight subclusters yielded no epidemiologic data, resulting in the closure of those clusters. The ninth subcluster, in the green box (right), contains the isolates associated with the restaurant cluster. Boldface indicates isolates from persons who ate at the restaurant; the star indicates the isolate from the food handler implicated in the outbreak.

By mid-December 2018, a total of 10 isolates had been confirmed as being related to this cluster: 3 isolates were initially identified through epidemiologic data and later linked by sequencing, and the other 7 were initially identified through sequencing and later confirmed to be associated with the restaurant through case-patient interview. By December 20, a total of 18 isolates had been genetically linked to this cluster through WGS. The additional 8 case-patients were either not available for additional follow-up (n = 5) or did not report having dined at this restaurant (n = 3). Case-patients associated with the restaurant had ordered food during November 2–4; of those, 10 had consumed raw lettuce and tomatoes in either a salad or sandwich, and 8 had consumed grilled chicken.

## Conclusion

Our study report illustrates that classic epidemiologic case follow-up integrated with molecular approaches to cluster detection expanded the scope of a restaurant-associated outbreak. Using PFGE data only, a total of 84 isolates were included in this cluster of *Salmonella* Enteriditis pattern JEGX01.0004, making it difficult to identify which case-patients were likely to have common exposures. Open communication between epidemiologists and laboratory personnel about epidemiologic and WGS data narrowed the scope of the investigation to a clade within the larger PFGE cluster’s phylogenetic tree, focusing investigative activities and improving the timeliness of control measure implementation. The real-time sequencing and analysis of all JEGX01.0004 isolates contributed to the identification of additional patients and helped identify the source for a foodborne outbreak. 

We followed stringent next-generation sequencing data processing and filtering thresholds implemented by PulseNet. In brief, we trimmed reads at Q30 and included genomes only if the average coverage was >20×. This method generates high-confidence results and has been adopted by PulseNet since early 2018 (*7*). 

The infected food handler was probably shedding *Salmonella* and, if hygiene practices were inadequate, could have contaminated ready-to-eat foods such as lettuce and tomatoes. Alternatively, a ready-to-eat food could have been the common source for all 18 cases in this cluster.

CDC has called for ongoing strengthening and support of state and local health departments to investigate and report outbreaks associated with foodborne disease as a necessary mechanism to reduce the burden of foodborne illness in the United States (*8*). In Massachusetts, cross-discipline collaboration among state and local health departments improved foodborne illness surveillance and response to a *Salmonella* Enteritidis outbreak associated with a restaurant. The cluster was epidemiologically identified within 2 weeks of exposure for the first 3 case-patients, inspection services were deployed to the restaurant the same day, and the probable source of contamination was confirmed by PFGE and WGS in <2 weeks. The prompt linking of epidemiologic and laboratory data and the implementation of in-state WGS and analysis improved public health surveillance capacity and timeliness of outbreak detection and control.
